# Effectiveness of international virtual training on biorisk management in the context of COVID-19

**DOI:** 10.3389/fpubh.2022.888097

**Published:** 2022-10-11

**Authors:** Shamsul Arfin Qasmi, Claire Standley, Saima Mohsin, Samreen Sarwar, Laila Malik, Fatima Aziz

**Affiliations:** ^1^Bahawalpur Medical and Dental College, Bahawalpur, Pakistan; ^2^Global Health Security, Georgetown University, Washington, DC, United States; ^3^The Panjwani Center for Molecular Medicine and Drug Research (PCMD), Karachi, Pakistan; ^4^Health Security Partner, Lahore, Pakistan; ^5^Agha Khan University, Karachi, Pakistan

**Keywords:** biorisk management, International Virtual Training (IVT), COVID-19, GHSA, biosecurity

## Abstract

**Introduction:**

The COVID-19 pandemic has resulted in enormous increases in laboratory activities to keep pace with diagnostic testing and research efforts. However, traditional training, technical assistance, and capacity-building approaches were disrupted by the travel and movement restrictions put in place to control the spread of the disease. To address the needs of laboratorians and managers to conduct laboratory activities safely and securely during the pandemic, a highly interactive virtual training (IVT) workshop on biorisk management during COVID-19 was conducted through active learning strategies that connected speakers with participants. The objective of the training was to increase the basic knowledge and standards of biosafety and biosecurity practices, risk assessment, and control measures with reference specifically to the context of the COVID-19 pandemic and apply a rigorous evaluation methodology to assess the effectiveness of the IVT. The training covered a broad range of topics and encompassed national to international guidelines.

**Methods:**

Participants were selected through official channels at the national level, focusing on institutions within Pakistan. The sessions included lectures from international experts in biorisk management concepts, and incorporated poll questions as well as pre- and post-tests and feedback on the speakers' knowledge and presentation skills, to increase interactivity. The pre- and post-test comprised similar multiple-choice questions and provided to every participant to ascertain the impact of the training on awareness and knowledge of biorisk management topics and concepts, and results were compared using paired *t*-tests. For feedback on the speakers, participants were asked to submit their ratings measured on a five-point Likert scale. The reliability of the Likert scale was estimated using Cronbach's alpha. Analyses were performed using Microsoft Excel and SPSS version 23.

**Results:**

In total, 52 individuals from different laboratories across Pakistan and Pakistani students from abroad (China) as well participated in at least one session of the IVT. The participants' pre- and post-test scores showed a significant increase in knowledge and awareness (*p* < 0.001). The obtained Cronbach's alpha score was >0.8, indicating high reliability of the generated feedback on the IVT approach and speakers.

**Conclusion:**

The IVT on biosafety and biosecurity in the context of the COVID-19 pandemic proved beneficial for laboratory professionals and could be a useful model to continue in the future for raising awareness and knowledge.

## Introduction

Since ancient times, infectious disease has been a known threat to mankind. Due to the re-emergence of novel infectious diseases, countries all over the world have continued to investigate infectious diseases in laboratory settings. The safe running of biomedical laboratories has an impact on public safety and security in addition to the lives and health of the experimental team working in the facility (1, 2). To minimize risks and provide a safe work environment, biorisk assessment is a critical tool for the evaluation of infectious pathogens in the laboratory (3).

The Global Health Security Agenda (GHSA) is a collaboration between 100 countries including international organizations, and non-governmental bodies to achieve the goal of a future free of infectious disease-related global health risks (4). The pandemic caused by severe acute respiratory syndrome-coronavirus-2 (SARS CoV-2) serves as a reminder of the importance of the threats and gaps to prevent, detect, and respond in time throughout the world (5). Despite significant regulations and stringent containment measures, countries continue to face health security threats posed by infectious diseases, whether unintentional, deliberate, or natural.

Biological materials are handled worldwide in laboratories for numerous genuine, justifiable, and legitimate purposes, where small and large volumes of live microorganisms are replicated, where cellular components are extracted, and many other manipulations were undertaken for purposes ranging from educational, scientific, medicinal, and health-related to mass commercial and/or industrial production. Among them, an unknown number of these biomedical facilities, large and small, work with dangerous pathogens or their products every day (6). However, despite advances in technology, the availability of more sophisticated instruments for laboratory use, and the availability of personal protective equipment, human error remains one of the most inherent factors at the origin of accidents (7). Inadvertent exposures to infectious agents in the laboratory, and associated laboratory-acquired infections, are more common in low and middle-income countries (8, 9). According to the WHO, dual-use research of concern (DURC) constitutes research that may be legitimately conducted for biomedical or other benefits, but which might also be misapplied to do harm. Recent studies have led to renewed attention to DURC, as well as a corresponding ongoing debate over the importance of Gain of Function (GoF) experiments (10). GoF experiments are those in which pathogens are manipulated in ways that result in an increase in the pathogen's transmissibility or pathogenicity, or ability to resist known countermeasures. Studies involving GoF may be scientifically useful, for example, to expand knowledge of pathogen evolution, and to assist in surveillance efforts for emerging diseases. However, it can also be catastrophic if the laboratories fail or if new knowledge is used to develop biological weapons (9).

During the COVID-19 epidemic in Pakistan, the healthcare system was overwhelmed. It was not easy to maintain and follow strict laboratory biosafety guidelines (10). It was very important to find ways to train laboratory workers without exposing them to the virus (11, 12). Laboratory biorisk assessment is the backbone of biorisk management according to the Laboratory biosafety manual, 4th edition, and is the basis for implementing effective mitigation strategies (13). During the pandemic, laboratory workers have encountered challenges, ambiguities, and, in some cases, controversies as they endeavored to enhance testing capabilities while maintaining the quality of laboratory operations (14, 15).

In early 2020, the COVID-19 pandemic resulted in restrictions on many types of in-person gatherings, including training. This led to a rapid rise in training courses and seminars that were instead delivered virtually, with the added benefit that these sessions could then be much more globally accessible (16, 17). The objective of the training was to help laboratory personnel including private and public laboratories in Pakistan to improve their skills in biorisk management in the context of the ongoing COVID-19 pandemic. A highly interactive virtual workshop on biorisk management was conducted through active learning strategies that connected speakers with participants. The impact of the training was thoroughly evaluated by developing poll questions, pre-/post assessments, and feedback surveys.

## Materials and methods

An International Virtual Training on Biorisk Management (Biosafety & Biosecurity) for life sciences and healthcare laboratory professionals in the light of the COVID-19 pandemic was developed. The virtual nature of the training took into account the restrictions related to in-person training and avoiding direct physical contact, while the content focused on the need for training in biorisk management in laboratories supporting SARS-CoV-2 diagnostics. This need was addressed by designing and developing a webinar series, which was conducted between 5 and 13 of April 2021. In total, there were seven sessions, every 3 h in duration. The program consisted of 16 speakers of international and national fame in Biorisk Management who delivered and contributed the same content on the following: (1) Introduction to National Biosafety and Biosecurity Policy - Classification of Biosafety Cabinets and Introduction to NS1/ANSI 49 Standards; (2) Advice on the use of masks in the context of COVID-19; (3) COVID-19 and Interim Biosafety Guidelines for Laboratory Workers; 4) Risk Assessment (gather information, evaluate the risks, and develop a risk mitigation strategy, control measures, and risk communication); (5) Diagnostic Testing for COVID- 19; (6) System Thinking Approaches (STA); (7) PPE Selection and Use including shipment and transportation of infectious agents in the epoch of COVID-19 according to CDC guidelines; (8) Occupational Health and Safety during pandemic and how to manage stress, and psychological effects of COVID-19 on lab staff; (9) Importance of Institutional oversight of research in the era of COVID-19; (10) Working in enhanced BSL-2 and BSL-3 with SARS-CoV-2; (11) Sanitation of facilities potentially contaminated with SARS-CoV-2; (12) Disinfection, Decontamination, Sterilization in the wake of COVID−19 for laboratory workers; (13) Surveillance, Reporting and referral of Specimens SARS-CoV-2; (14) Challenges of biosecurity and its importance in the recent pandemic; (15) Biological waste management in the context of COVID-19; (16) Biological waste management in the light of COVID-19; (17) Emergency preparedness in COVID−19. To evaluate laboratory biosafety and biosecurity knowledge in Pakistan the risk assessment was done, and topics were selected in the light of the current situation of the COVID-19 pandemic to prevent laboratory-acquired infections when incidents of COVID-19 were rising in Pakistan.

The participants were selected through proper advertisement using social media platforms and organizational emails. Evaluation of the interactive virtual training (IVT) included the use of poll questions pre- and post-assessment tests before and after the training, consisting of multiple-choice questions administered to the participants, and feedback from the participants, measured using a Likert scale, regarding the speakers' knowledge and presentation skills as well as their impressions of the training overall. The comparison among different variables was analyzed through appropriate tables, graphs, and percentages. Pre- and post-test scores were compared using paired *t*-tests at 95% CI. Reliability and consistency of feedback from the participants on the speakers and overall training were evaluated by using Cronbach's alpha. Statistical analyses were performed using Microsoft Excel (Microsoft Corporation, Redmond) and SPSS version 23 (IBM, Armonk).

## Results

The participants' sociodemographic information (Table 1) shows that a total of 52 participants enrolled to attend the webinars, out of which 30 (58%) were men and 22 (42%) were women.

**Table 1:** Participants' sociodemographic information.

**Variables**	***n***	**Percentage (%)**
** Gender**		
Male	30	57.69%
Female	22	42.31%
** Participants' Institute Jurisdiction**		
From Balochistan	1	1.92%
From Baltistan	3	5.77%
From KPK	8	15.38%
From Punjab	13	25.00%
From Sindh	25	48.08%
From Overseas (China)	2	3.85%

**Figure 1:**
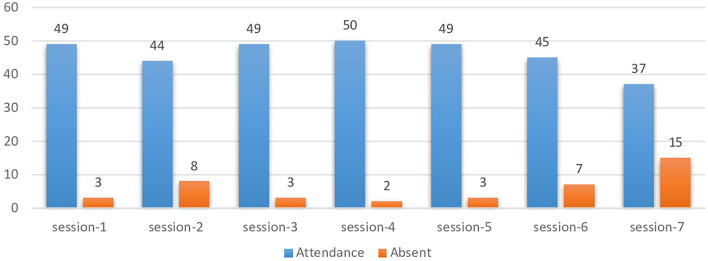
Participant's attendance in session.

The weekly attendance of 52 participants is shown in Figure 1. While the exact numbers of attendance varied from week to week, overall, 37 participants attended all seven sessions, and the average attendance was 46 persons throughout the sessions.

Pre-test assessments at the beginning of the webinars and post-test assessments at the end of the webinars were conducted to ascertain the impact on participant awareness of the key topics. Only 36 (64%) participants out of 52 completed the pre- and post-assessments. As shown in Table 2A. The major difference in mean test scores of pre- and post-assessment results was observed to increase from 18.69 to 24 and their mean difference was −5.31. The correlation among pre- and post-tests scores was 0.71, showing that there was a moderate positive (uphill) linear relationship. The variation between both variables was around 50.41%.

Table 2Pre-test and post-test score evaluation.

**Table:** 

**A**			
**Paired samples statistics**			
	**Mean**	***N***	**Std. deviation**
Pre_Test_Score	18.69	36	0.560
Post_Test_Score	24.00	36	0.676
Paired samples correlations			
	***N***	**Correlation**	**Sig**.
Pre_Test_Score & Post_Test_Score	36	0.710	0.000

**Table:** 

**B**								
**Paired samples test**								
	**Paired differences**					**t**	**df**	**Sig. (2-tailed)**
	**Mean**	**Std. deviation**	**Std. error Mean**	**95% confidence** **interval of** **the difference**	
				**Lower**	**Upper**			
Pre_Test_Score -Post_Test_Score	−5.306	2.896	0.483	−6.286	−4.326	−10.990	35	0.000

The level of significance was determined using paired *t*-test with 95% CI that showed a highly significant *P*-value (*P* < 0) that is representing there is a significant difference between tested variables, i.e., pre-test score and post-test score (Table 2B).

To ensure the validity of the results and with the intention to improve the quality of the webinars in the future. The participants' responses/feedback regarding all the presented IVT webinars by different speakers are also evaluated and their ratings were measured on a five-point Likert scale (1-Strongly disagree, 2-Disagree, 3-Neutral, 4-Agree, and 5-Strongly agree) shown in Table 3. The reliability of the Likert scale was estimated using Cronbach's alpha, which showed all variables to have a maximum score >0.8 (0.946), indicating the high reliability of the generated feedback evaluation (Table 4).

**Table 3:** Overall participant's feedback.

**Item statistics**						
**Feedback questions**	**Neutral**	**Agree**	**Strongly agree**	**Mean**	**Std. deviation**	**Cronbach's alpha if item deleted**
The training objectives were clear to me	1	9	29	4.72	0.510	0.866
I will be able to use what I learned in this virtual training	2	13	24	4.56	0.598	0.867
This training was a good way for me to learn about Bio risk management	1	10	28	4.69	0.521	0.866
The instructors were knowledgeable	2	15	22	4.51	0.601	0.864
The instructors were well prepared	0	8	31	4.79	0.409	0.872
The instructors were helpful and responsive to questions	1	5	33	4.82	0.451	0.877
The pace of this training was appropriate	2	15	22	4.51	0.601	0.864
This training lived up to my expectations	0	14	25	4.64	0.486	0.866
The training content was. [Relevant]	0	9	30	4.77	0.427	0.883
The training content was. [Easy to understand]	2	15	22	4.51	0.601	0.868
The training content was: (Comprehensive)	1	14	24	4.59	0.549	0.870

**Table 4:** Cronbach's alpha reliability score on speakers' evaluation.

**Reliability statistics**	
**Cronbach's alpha**	***N*** **of speakers**
0.946	16

## Discussion

Biorisk management is a major problem that has been overlooked at various stages of graduate education, research training, and laboratory professional skill development in the context of Pakistan (18). In a past study, we highlighted the significance of biosafety and biosecurity protocols and policies (18). Thus, all laboratory professionals should have a basic knowledge of standard microbiological practices, risk assessment, and control measures (19).

Infections in laboratories not only threaten the health of laboratory workers, but they can also result in the unintentional release of organisms into the wider environment or community (19). A major gap has been seen in implementing biorisk management in laboratories due to a lack of awareness in Pakistan (20). These gaps can be addressed through educational initiatives on biosafety and biosecurity.

The online training program on “Biorisk Management in context of COVID-19” was very successful as confirmed by the increased average scores in the post-training evaluation and feedback survey questionnaire. This proves that virtual biosafety and biosecurity training program has significant importance in the recent pandemic and afterward. Participants shared their online training experience at the end of the webinar series and showed their interest in hybrid training programs including in-person to gain more hands-on training in the future.

This training course also identified several other challenges and gaps in developing and implementing resilient biosafety capacity-building programs. These challenges and gaps have been identified through discussion among participants. To ensure safe and secure conditions, laboratories must implement a comprehensive biorisk management system that fulfills the requirements of GHSA Action Package 3 (Biosafety and Biosecurity) and bioethical guidelines^1^. Recommendations were also received from participants in the feedback questionnaire.

In this workshop participants also discussed similarities and differences in the infrastructure and training associated with BSL-2 and BSL-3 laboratories. Participants also showed consensus that hands-on training, as well as didactic training, are essential for developing and implementing a researcher's competence to work in a high-containment facility.

Individuals who participated in the workshop also highlighted the training of laboratory professionals on risk assessment. These biosafety training programs should be flexible and adapted according to the target research facility, research area, and personnel working there as what may be appropriate to one context may not be suitable to another: one size does not fit all. In addition, effective awareness of biorisk management is still required, as well as resources and expertise for the successful implementation of biosafety and biosecurity at the national level. The biotechnology sector is continuously growing in Pakistan. Therefore, training on biorisk management should also be leveraged to sensitize the scientific community on dual-use research issues, which is a neglected area in Pakistan (21).

## Conclusion

The recommendations that were received from participants during this IVT are important to properly fill the existing gaps in biosafety and biosecurity in Pakistan. Biosafety and biosecurity training are of utmost significance in the current challenging situation of the COVID-19 pandemic. The findings of this project highlight the raising awareness of biorisk measures in public and private laboratories. Increasing knowledge on biorisk management can serve to reduce the risk of intentional or unintentional release of pathogens, thus improving the safety of laboratory workers, the community, and the environment. However, to continue with didactic training on risk assessment, it is observed that support from the public and private sectors at national and international levels will have an additional impact on the implementation of biorisk management.

## Data availability statement

The original contributions presented in the study are included in the article/supplementary material, further inquiries can be directed to the corresponding author.

## Ethics statement

The studies involving human participants were reviewed and approved by Departmental Bioethics Committee, Bahawalpur Medical and Dental College, Bahawalpur, Pakistan. The patients/participants provided their written informed consent to participate in this study.

## Author contributions

SQ: conceptualization, methodology, writing, reviewing, and editing. FA: writing of the original draft, reviewing, and editing. SM: writing of the first draft conclusion. LM: statistical analysis and interpretation of the data. SQ, FA, and CS: critical review. SS: project administration, resources, and supervision. All authors contributed to the article and approved the submitted version.

## Funding

This work was supported by Health Security Partners, USA. We also thank our trainers/facilitators, without whom this work would not have been possible.

## Conflict of interest

The authors declare that the research was conducted in the absence of any commercial or financial relationships that could be construed as a potential conflict of interest. The reviewers AA and SF declared a shared affiliation with the author SM to the handling editor at the time of review.

## Publisher's note

All claims expressed in this article are solely those of the authors and do not necessarily represent those of their affiliated organizations, or those of the publisher, the editors and the reviewers. Any product that may be evaluated in this article, or claim that may be made by its manufacturer, is not guaranteed or endorsed by the publisher.

## Footnotes

1 https://www.cdc.gov/globalhealth/security/actionpackages/default.htm
